# Supplementary material for: Arabidopsis nicotianamine synthases comprise a common core-NAS domain fused to a variable autoinhibitory C terminus
Source: J Biol Chem. 2023 Apr 21;299(6):104732. doi: 10.1016/j.jbc.2023.104732 (PMC10248798; doi:10.1016/j.jbc.2023.104732)
Supplement: Supporting Figures S1–S10 and Tables S2, S4 and S6 [file mmc5.pdf]

Arabidopsis nicotianamine synthases comprise a common core-NAS domain fused to a variable autoinhibitory C terminus

Hiroyuki Seebach, Gabriel Radow, Michael Brunek, Frank Schulz, Markus Piotrowski and Ute Krämer

Supporting Information

Figure S1. Reactions catalyzed by Nicotianamine Synthase (NAS) and NAS-like proteins (included in this file)
Figure S2. Activities of purified Methylthioadenosine Nucleosidase (MtnN) and Adenine Deaminase (AdeD) in NAS reaction buffer (included in this file)
Figure S3. Detection of nicotianamine by mass spectrometry (included in this file)
Figure S4. Activities of purified AtNAS1 and AtNAS1 mutants (included in this file)
Figure S5. One-pot biosynthesis of nicotianamine from L-methionine and ATP (included in this file)
Figure S6. Unrooted phylogenetic tree representation supporting Figure 4 (included in this file)
Figure S7. *NAS* genes of the mosses *P. patens* and *C. purpureus* and of the fungi *N. crassa*, *M. oryzae* and *D. ampelina* all contain an intron at a conserved position (included in this file)
Figure S8. Alignment of amino acid sequences of C-terminal regions of long NAS proteins from angiosperm plants (included in this file)
Figure S9. NAS isoforms in the Brassicaceae family of dicotyledonous angiosperms (included in this file)
Figure S10. Linker region in AtNAS and CntL (included in this file)
Figure S11. Settings used in Mass Spectrometry method (*.pdf)

Table S1. All NAS and NAS-like proteins used in the phylogenetic analysis (*.xlsx)
Table S2. List of bacterial strains and plasmids (included in this file)
Table S3. List of primers (*.xlsx)
Table S4. PCR conditions (included in this file)
Table S5. DNA sequences of *NcNAS*, *PpNAS* and *MetrNAS2* codon-optimized for *E. coli* (*.xlsx)
Table S6. Calculated molar mass for proteins used in this study (included in this file)

Data S1. Multiple sequence alignment of NAS and NAS-like proteins (*.txt)

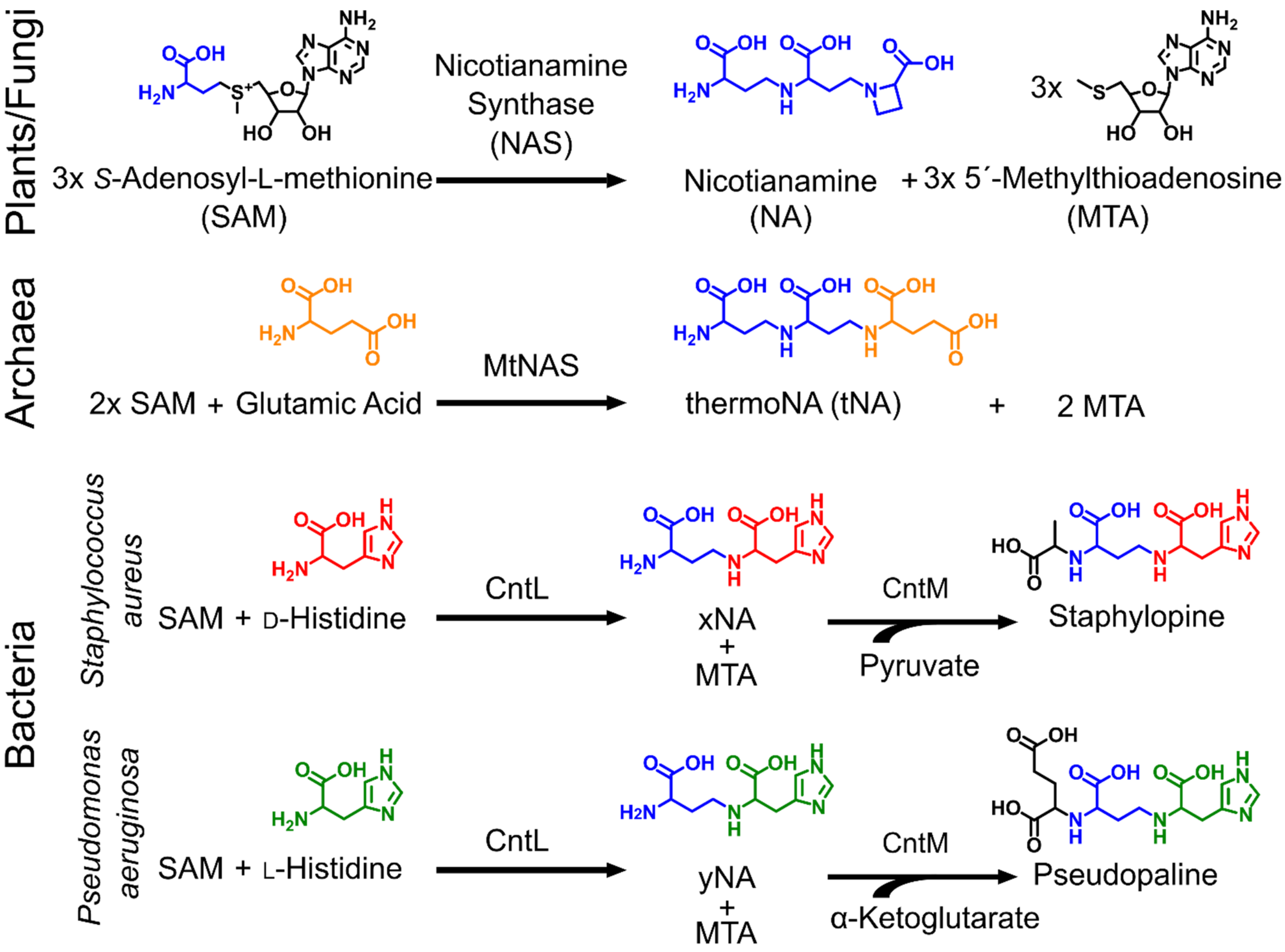

Figure S1. Reactions catalyzed by Nicotianamine Synthase (NAS) and NAS-like proteins.
NAS of plants and fungi utilize the α-aminobutyrate group (blue) of each of three *s*-adenosylmethionine (SAM) molecules to form one molecule of nicotianamine (NA) and three methylthioadenosine (MTA) molecules. Known NAS-like proteins of archaea appear to use one glutamate (orange) and the α-aminobutyrate groups of two SAM molecules to form one thermo-nicotianamine (tNA) and two MTA molecules. The characterized bacterial NAS-like proteins (CntL) use one D-/L-histidine (red/green) and one α-aminobutyrate group from SAM to form one xNA/yNA and MTA. Subsequently, Opine Dehydrogenase (CntM) utilizes NADPH and an α-keto acid to convert xNA with pyruvate to staphylopine, or yNA with α-ketoglutarate to pseudopaline. NAS and the NAS-like MtNAS are thought to catalyze the formation of their products from the substrates sequentially in a step-wise manner inside a central reaction cavity of the protein, from the right to the left of the products shown here.

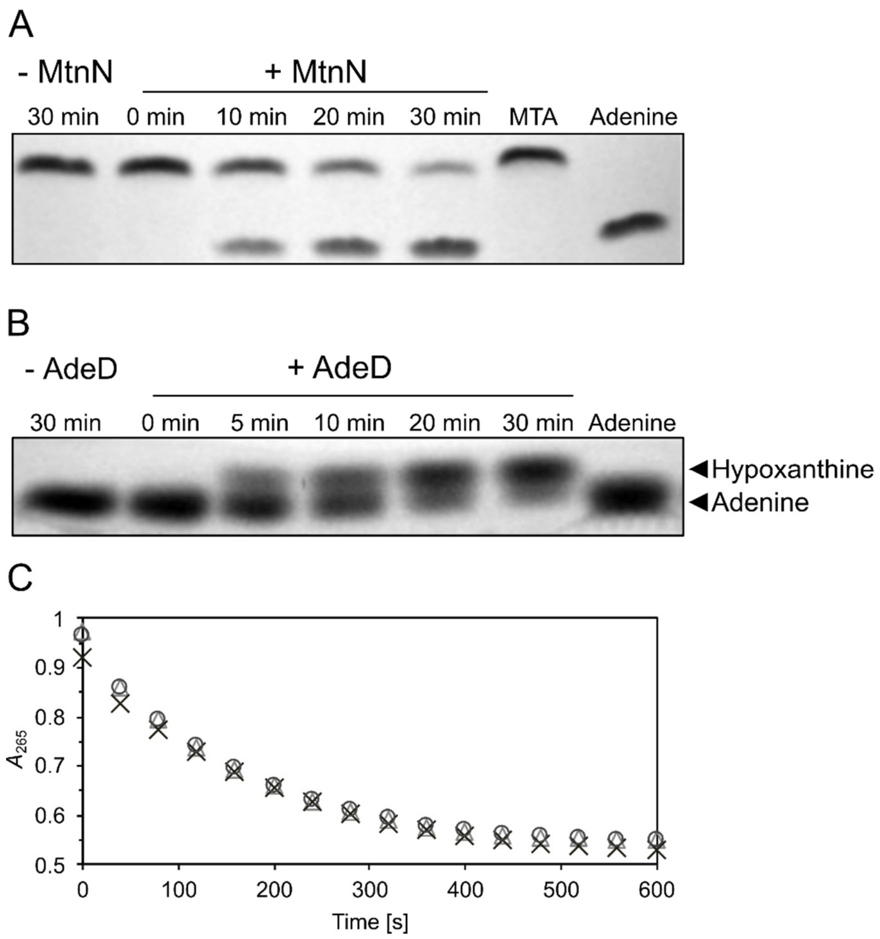

Figure S2. Activities of purified Methylthioadenosine Nucleosidase (MtnN) and Adenine Deaminase (AdeD) in NAS reaction buffer.
(A, B) MtnN (A) and AdeD (B) were incubated with their respective substrate in NAS reaction buffer (50 mM Tris/HCl, pH 8.7), and the reaction products were separated by thin-layer chromatography and visualized under UV light at different time points after the start of the reaction (0 min). (C) Photometric measurement over time of the coupled conversion of MTA to hypoxanthine catalyzed by the enzymes MtnN and AdeD in combination in NAS reaction buffer. Square, triangle and diamond each represent one technical replicate. MTA was added at time point 0 s.

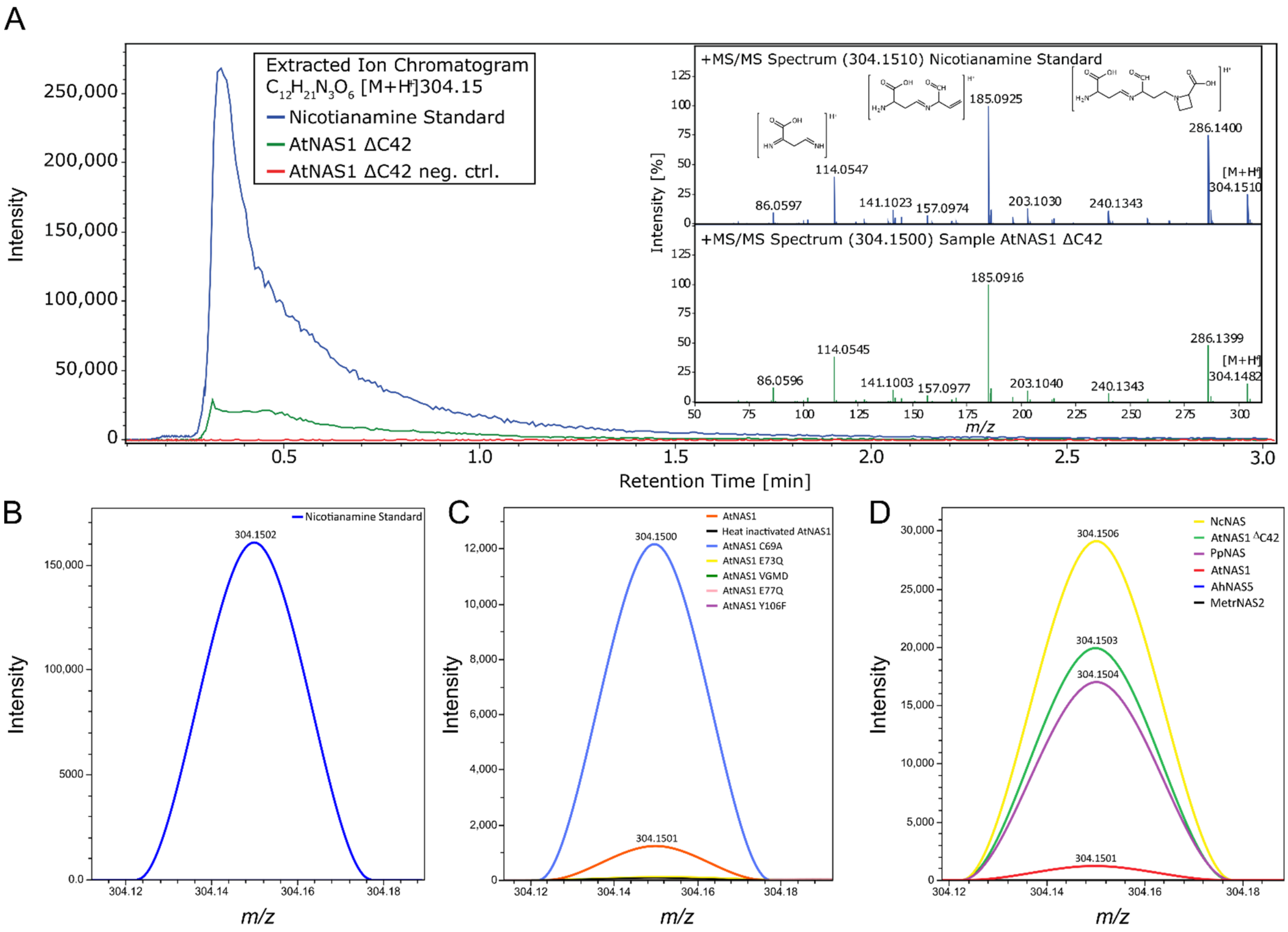

Figure S3. Detection of nicotianamine by mass spectrometry.
Purified NAS proteins (0.15 mg protein ml^{-1}) were incubated with MtnN (1 mg protein ml^{-1}) and 3 mM SAM in a total volume of 30 µL at 30°C for 2 h before LC-MS. HRMS was carried out in positive ion mode (for details see SI Fig. S11). (A) Extracted ion chromatograms (*m*/*z* 304.15) for nicotianamine standard and reaction products obtained with active or heat-denatured (neg. ctrl.) AtNAS1 ΔC42. The inset shows the MS/MS spectra for nicotianamine including probable fragment structures, and for *m*/*z* 304.15 in the reaction products obtained with active AtNAS1 ΔC42. (B-D) Intensities of nicotianamine peaks for the nicotianamine standard (B), for reaction products of AtNAS1 (red) and AtNAS1 variants C69A (blue), E73Q (yellow), ΔVGMD (green), E77Q (pink) and Y106F (violet)(C), as well as of NcNAS (yellow), AtNAS1ΔC42 (green), PpNAS (violet), AtNAS1 (red), AhNAS5 (blue) and MetrNAS2 (black)(D).

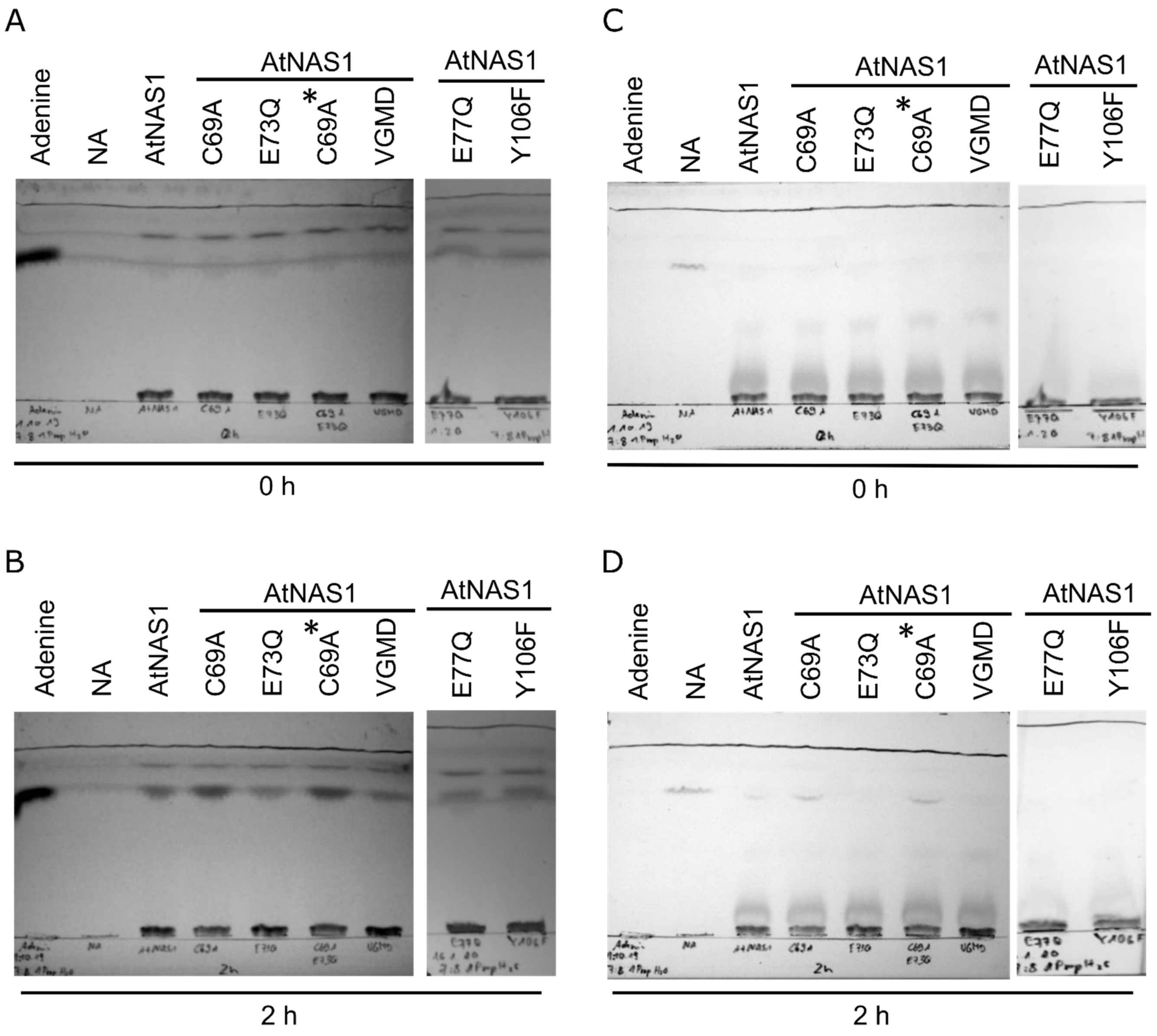

Figure S4. Activities of purified AtNAS1 and AtNAS1 mutants.
AtNAS1 and AtNAS1 mutants (4.5 µg) and MtnN (10 µg) were co-incubated in the presence of SAM (5 mM) in a total volume of 30 µl. The reaction was started by incubating the mixture at 30°C, and it was stopped immediately (0 h, A and C) or 2 h (B and D) after the start of reaction by flash-freezing. Aliquots of 5 µl per reaction were separated by TLC, and the products were visualized by UV light (A and B) or ninhydrin staining (C and D). NA: nicotianamine. AtNAS1 C69A was unintentionally loaded twice (see *). AtNAS1 E77Q and AtNAS1 Y106F were run on a different TLC plate under identical conditions. Cropped images were taken from the images shown here and assembled to generate Fig. 3C.

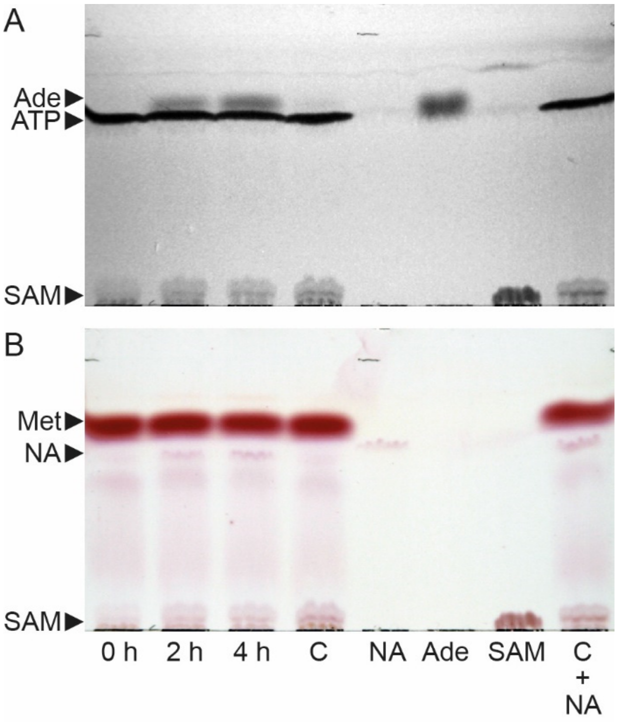

Figure S5. One-pot biosynthesis of nicotianamine from L-methionine and ATP.
(A, B) AtNAS1 (42 µg), MetK (60 µg) and MtnN (12 µg) where co-incubated in the presence of L-methionine (Met, 10 mM) and ATP (10 mM) in a total volume of 120 µl at 30°C for up to 4 h. At the indicated time points, 5-µL aliquots of the reaction mix were separated by TLC, and compounds were visualized by UV light (A) and ninhydrin staining (B). Adenine (Ade, 16 nmol standard), nicotianamine (NA, 8 nmol standard) and *S*-adenosylmethionine (SAM, 25 nmol standard) were run as standards. C: Negative control (conducted with heat-denatured AtNAS1), C + NA: As C, but with added nicotianamine (192 nmol). After 4 h the amount of adenine in the 5-µl volume loaded was approximated to be ~8 nmol (about half of the amount present in the adenine standard, Ade). This corresponded to a total amount of 192 nmol adenine produced in the full 120-µl reaction volume which was equivalent to 64 nmol nicotianamine.

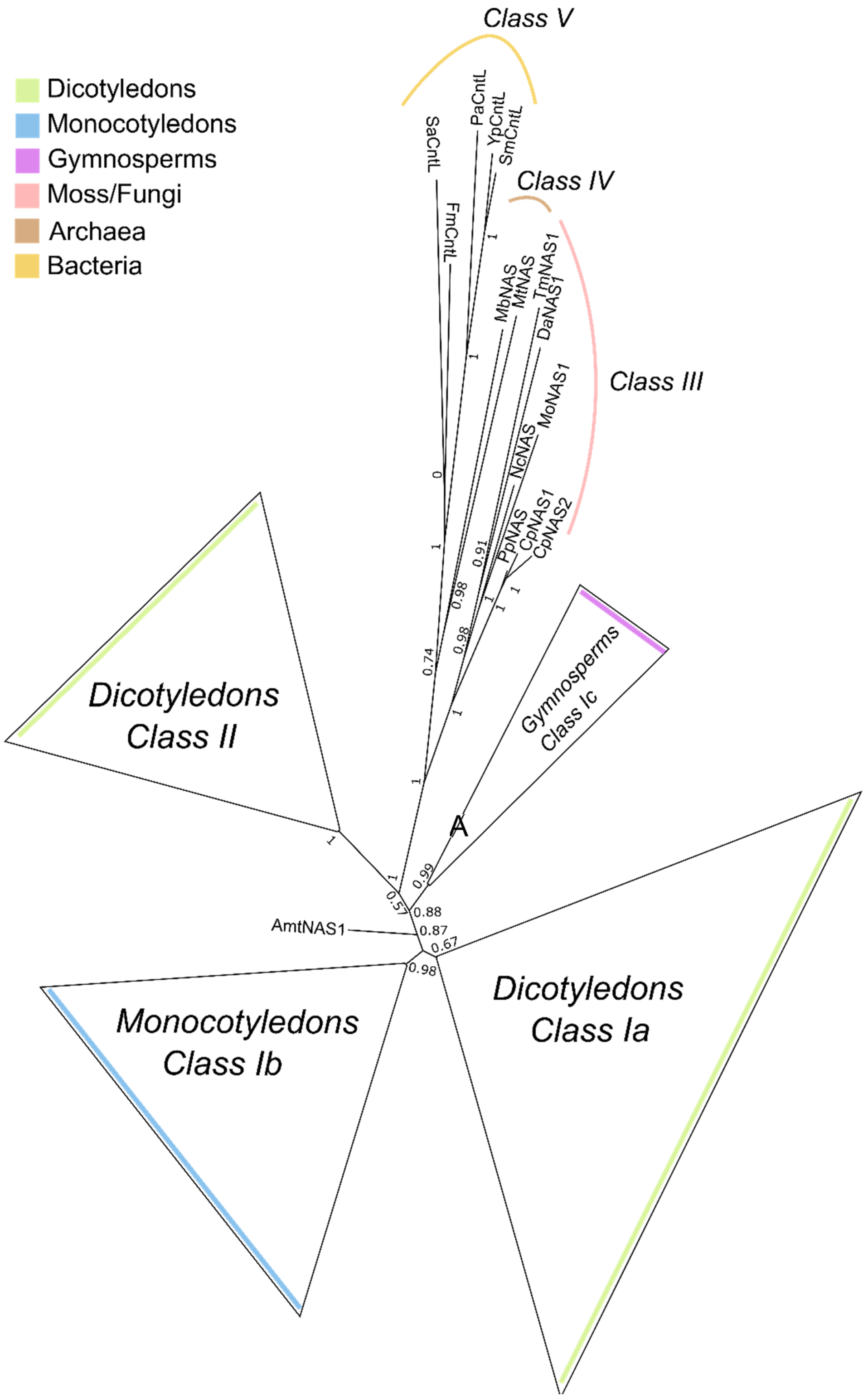

Figure S6. Unrooted phylogenetic tree representation supporting Figure 4.
Phylogenetic tree of NAS proteins from seed plants, mosses, and fungi, as well as NAS-like proteins from bacteria and archaea (SI Table S1) obtained by Bayesian inference. For a better visualization branches for NAS from plants were collapsed into groups according the NAS classes Ia, Ib, Ic and II. Numbers at branch positions reflect clade credibility values between 0.52 and 1.

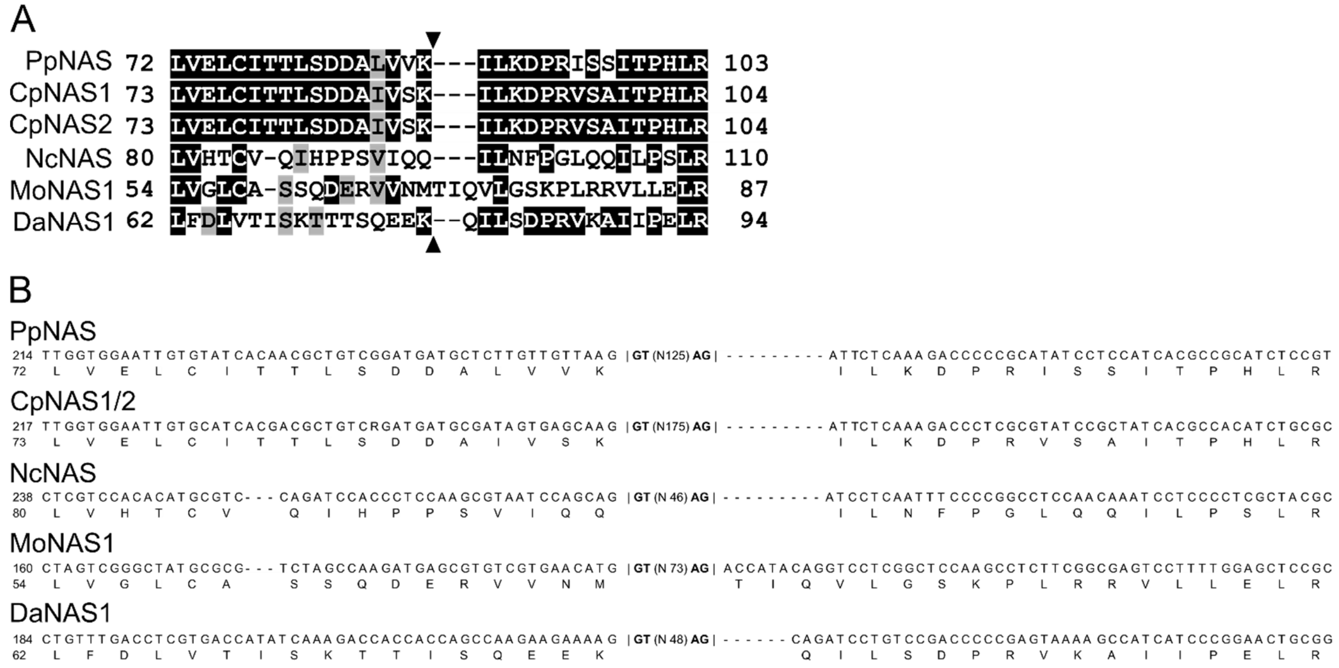

Figure S7. *NAS* genes of the mosses *P. patens* and *C. purpureus* and of the fungi *N. crassa*, *M. oryzae* and *D. ampelina* all contain an intron at a conserved position.
(A) To visualize the conserved position of the intron, part of an amino acid alignment is shown. The intron position is indicated by triangles. (B) Genomic sequence (upper rows) alongside amino acid sequence (bottom rows) indicated centrally within each codon of the respective *NAS* gene. The sequences are aligned as in (A). In all 6 sequences, the intron is in phase 0 (intron position given by vertical lines; N followed by a number specifies the length of each intron, and only the initial and final two nucleotides of each intron are specified). The *NAS* gene of *M. oryzae* contains a second intron downstream in its sequence. Amino acids are shown on a black/grey background whenever ≥ 50% of them are identical/similar. Similarity groups were based on the default classification of MultipleAlignShow (ILV, FWY, KRH, DE, GAS, P, C, TNQM)(55). Pp: *Physcomitrella patens* (Pp3c8_970), CepurR40: *Ceratodon purpureus* male isolate R40 (CepurR40.10G154600), CepurGG1: *C. purpureus* female isolate GG1 (CepurGG1.10G14960), Nc.: *Neurospora crassa* (XP_958379.1), Mo: *Magnaporthe oryzae* (XP_003719353.1), Da: *Diaporthe ampelina* (KKY38707.1).

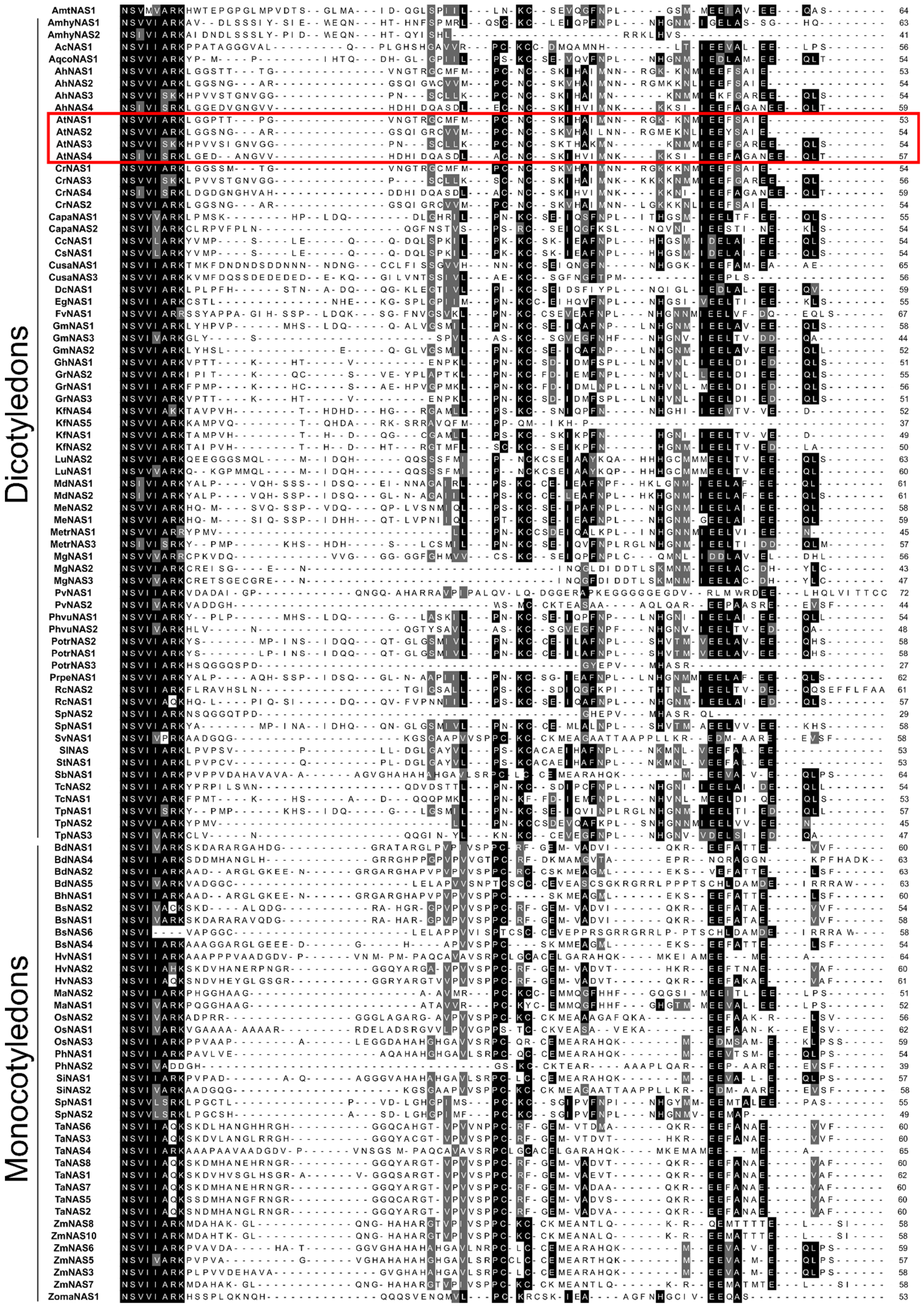
Dicotyledons
Monocotyledons

Figure S8. Alignment of amino acid sequences of C-terminal regions of long NAS proteins from angiosperm plants.
Amino acids are shown on a black/grey background whenever ≥ 50% of sequences were identical/similar, considering only the sequences ungapped at the respective position. Similarity groups were based on the default classification of MultipleAlignShow (ILV, FWY, KRH, DE, GAS, P, C, TNQM)(55). The alignment was carried out in MegaXI using ClustalW with standard settings. *A. thaliana* NAS1 to NAS4 are boxed in red. For abbreviations of species names see SI Table S1.

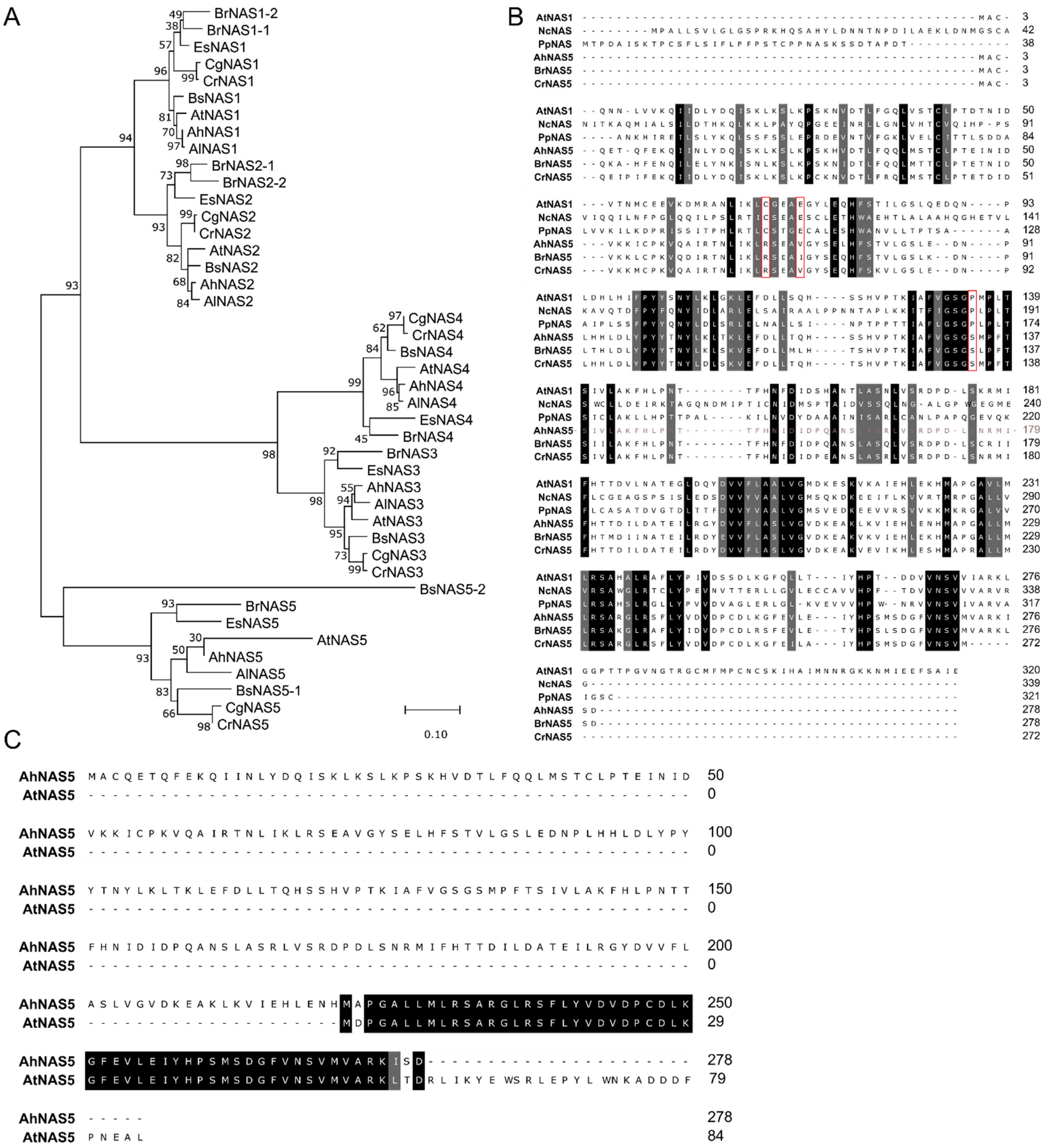

Figure S9. NAS isoforms in the Brassicaceae family of dicotyledonous angiosperms.
(A) Maximum likelihood phylogenetic tree of selected NAS sequences from the Brassicaceae. The existence of 5 paralogous NAS proteins in the Brassicaceae is evident. The percentage of trees in which the associated taxa clustered together is shown next to the branches. Branch lengths reflect the number of substitutions per site, scaled as indicated below the tree. (B) Full amino acid sequence alignment of selected NAS proteins observed to exhibit enzyme activity here (NcNAS, PpNAS, AtNAS1) and NAS5 proteins from *A. halleri* (no detectable enzyme activity; see SI Fig. S6), *B. napus* and *C. rubella*. Amino acids are shown on a black/grey background whenever ≥ 50% of them are identical/similar. Similarity groups were based on the default classification of MultipleAlignShow (ILV, FWY, KRH, DE, GAS, P, C, TNQM)(55). Red boxes highlight amino acids conserved among active NAS proteins which are divergent in NAS5 proteins of the Brassicaceae. (C) Full amino acid sequence alignment of NAS5 proteins from *A. halleri* and *A. thaliana. AtNAS5* (AT4G26483) is a pseudogene encoding merely a short fragment of the NAS5 protein.

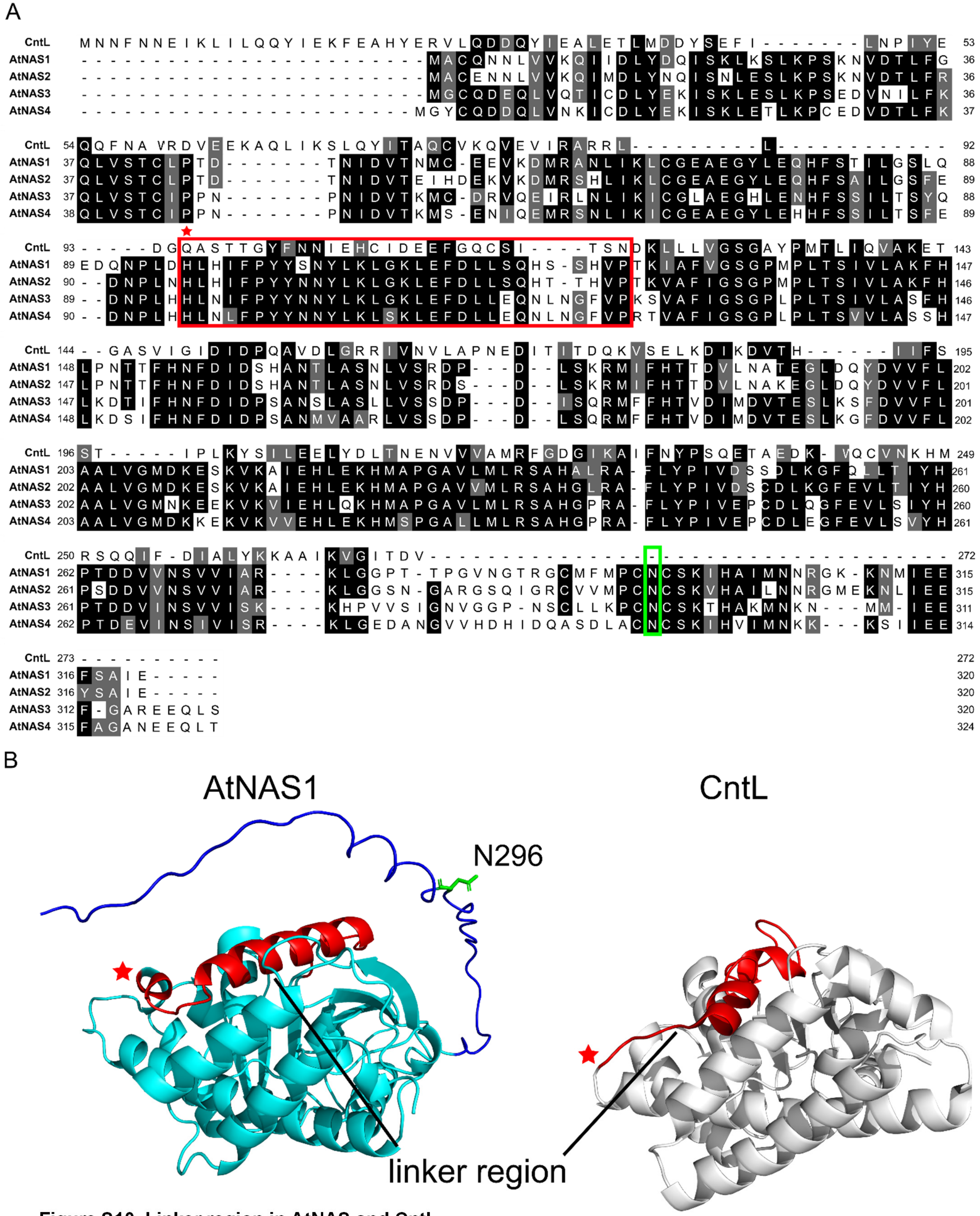

Figure S10. Linker region in AtNAS and CntL.
(A) Full amino acid alignment of AtNAS1-4 and *Staphylococcus aureus* CntL. Red/green boxes mark the amino acids corresponding to the conformationally dynamic linker region of CntL (43)/N296 contributing to auto-inhibition of AtNAS1 (see Fig. 6). Amino acids are shown on a black/grey background whenever ≥ 50% of them are identical/similar. Similarity groups were based on the default classification of MultipleAlignShow (ILV, FWY, KRH, DE, GAS, P, C, TNQM)(55). (B) Modified protein model of AtNAS1 (left, generated by alphafold 2) and CntL (right, PDB:7C9M), with red/green used to identify regions as defined in (A) (44). The elongated C-terminus of AtNAS1 is colored in dark blue.

Table S2. List of bacterial strains and plasmids.	
**Bacterial strain**	**Supplier**
XL1-Blue	Agilent Technologies, Santa Clara, US
BL21-CodonPlus (DE3)-RIL	Agilent Technologies, Santa Clara, US
BL21 DE3 pLysS	EMD Millipore, Burlington, US
**Plasmid**	**Supplier**
pGEM®-T Easy	Promega, Madison, US
pET101 Directional TOPO ™	ThermoFisher Scientific, Waltham, US
pET-21b (+)	EMD Millipore, Burlington, US
pMA-RQ	ThermoFisher Scientific, Waltham, US

Table S4. PCR conditions.

Amplicon	Encoded Protein	PCR conditions
mtnN	MtnN	95 °C 3 min, 30 × [95 °C 30 s, 70 °C 30 s, 72 °C 60 s], 72 °C 10 min
adeD	AdeD	98 °C 3 min, 30 × [98 °C 10 s, 68 °C 30 s, 72 °C 60 s], 72 °C 5 min
metK	MetK	95 °C 3 min, 30 × [95 °C 30 s, 66 °C 30 s, 72 °C 60 s], 72 °C 10 min
MTH675	MtNAS	98 °C 30 s, 30 × [98 °C 10 s, 72 °C 60 s], 72 °C 5 min
AT5G04950	AtNAS1	94 °C 10 min, 35 × [93 °C 30 s, 52 °C 30 s, 72 °C 2 min], 72 °C 90min
	AtNAS1 C69A	95 °C 2 min, 20x [98 °C 20 s, 55 °C 30 s, 72 °C 3,5 min], 72 °C 5 min
	AtNAS1 E73Q	95 °C 2 min, 20x [98 °C 20 s, 68 °C 30 s, 72 °C 3,5 min], 72 °C 5 min
	AtNAS1 VGMD	95 °C 2 min, 20x [98 °C 20 s, 52 °C 30 s, 72 °C 3,5 min], 72 °C 5 min
	AtNAS1 E77Q	95 °C 30 s, 18x [98 °C 10 s, 55 °C 10, 72 °C 3,5 min], 72 °C 5 min
	AtNAS1 Y106F	95 °C 30 s, 18x [98 °C 10 s, 55 °C 10, 72 °C 3,5 min], 72 °C 5 min
	AtNAS1 N296D	95 °C 30 s, 18x [98 °C 10 s, 55 °C 10, 72 °C 3,5 min], 72 °C 5 min
AT5G56080	AtNAS2	98 °C 30 s, 30 × [98 °C 10 s, 64 °C 15 s, 72 °C 30 s], 72 °C 5 min
	AtNAS2 ΔC43	98 °C 30s, 30 × [98 °C 10 s, 53,8 °C 10 s, 72 °C 30 s], 72 °C 10 min
AT1G09240	AtNAS3	98 °C 30 s, 30 × [98 °C 10 s, 64 °C 15 s, 72 °C 30 s], 72 °C 5 min
	AtNAS3 ΔC42	98 °C 30s, 30 × [98 °C 10 s, 53,8 °C 10 s, 72 °C 30 s], 72 °C 10 min
AT1G56430	AtNAS4	94 °C 10 min, 35 × [93 °C 30 s, 52 °C 30 s, 72 °C 2 min], 72 °C 90min
	AtNAS4 ΔC47	98 °C 30s, 30 × [98 °C 10 s, 53,8 °C 10 s, 72 °C 30 s], 72 °C 10 min
	AtNAS1 ΔC13	98 °C 30s, 30 × [98 °C 10 s, 70 °C 25 s, 72 °C 30 s], 72 °C 10 min
	AtNAS1 ΔC22	98 °C 30s, 30 × [98 °C 10 s, 67,2 °C 15 s, 72 °C 30 s], 72 °C 10 min
	AtNAS1 ΔC31	98 °C 30s, 30 × [98 °C 10 s, 67,2 °C 15 s, 72 °C 30 s], 72 °C 10 min
	AtNAS1 ΔC34	98 °C 30s, 30 × [98 °C 10 s, 72 °C 30 s], 72 °C 10 min
	AtNAS1 ΔC42	98 °C 30s, 30 × [98 °C 10 s, 72 °C 30 s], 72 °C 10 min
	AtNAS1 ΔC43	98 °C 30s, 30 × [98 °C 10 s, 72 °C 30 s], 72 °C 10 min
	AtNAS1 ΔC44	98 °C 30s, 30 × [98 °C 10 s, 72 °C 30 s], 72 °C 10 min
	AtNAS1 ΔC45	98 °C 30s, 30 × [98 °C 10 s, 72 °C 30 s], 72 °C 10 min
	AtNAS1 ΔC46	98 °C 30s, 30 × [98 °C 10 s, 72 °C 30 s], 72 °C 10 min

Table S6. Calculated molar mass for proteins used in this study.

**Protein**	**molar mass [Da]**
AtNAS1	35,547
AtNAS1 ΔC42	30,936
MtnN	24,398
AdeD	63,739
MetK	41,952
NcNAS	36,928
PpNAS	34,505
MetrNAS2	31,978
AtNAS2	35,679
AtNAS3	35,751
AtNAS4	36,352
AtNAS2 Δ43	31,004
AtNAS3 Δ42	31,201
AtNAS4 Δ47	31,203
AtNAS1 ΔC13	34,069
AtNAS1 ΔC22	32,991
AtNAS1 ΔC31	31,974
AtNAS1 ΔC34	31,659
AtNAS1 ΔC43	30,878
AtNAS1 ΔC44	30,821
AtNAS1 ΔC45	30,708
AtNAS1 ΔC46	30,580
AtNAS1 N296D	35,548

https://web.expasy.org/protparam/
