# Supplementary material for: Arabidopsis nicotianamine synthases comprise a common core-NAS domain fused to a variable autoinhibitory C terminus
Source: J Biol Chem. 2023 Apr 21;299(6):104732. doi: 10.1016/j.jbc.2023.104732 (PMC10248798; doi:10.1016/j.jbc.2023.104732)
Supplement: Supporting Figure S11 [file mmc6.pdf]

Settings used in Mass Spectrometry method.

Method Set: D:\Methods\Susanna\110-1300 autoMSMS pos_.m

BRUKER

otofControl

General Information

**Method Name:**	110-1300 autoMSMS pos_.m	**Saved:**	2018/09/05 09:38:08+02:00
**Application Name:**	Bruker otofControl	**Application Version:**	4.1.3.5
**Device Type:**	compact	**Device Serial Number:**	8255754.20147
**Operator:**	Demo User	**Host:**	COMPACT-20147
**Operating System:**	Windows 7 Professional	**Organisation:**	Bruker Daltonik GmbH

Chromatogram

Chromatogram Traces

Enabled	Color	Type	Masses	Width	Polarity	Filter
On	Red	BPC			±	MS
On	Blue	TIC			±	MS
On	Black	TIC			±	All MS/MS

SPL

**Scheduled Precursor List:**	Off

Segment 1

0 .... 0.02 min

Main

**Polarity:**	Positive	**Scan Mode:**	MS
**Mass Range from:**	110 m/z	**Mass Range to:**	1300 m/z
**Rolling Average:**	Off	**Rolling Average No.:**	2
**Spectra rate:**	8.00 Hz	**View:**	Expert

Mode

**Save Spectra:**	Line and Profile Spectra	**Line Spectra Calculation:**	Use Maximum Intensity
**Absolute Threshold (per 1000 sum.):**	25 cts.	**Peak Summation Width:**	3 pts.
		**Focus Active:**	Off
**Mark as Calibration Segment:**	Off		

Source

**Source:**	ESI		
**End Plate Offset:**	500 V	**Capillary:**	4500 V
**Nebulizer:**	2.2 Bar	**Dry Gas:**	10.0 l/min
**Dry Temp:**	220 °C	**Divert Valve:**	Waste 1-6

Tune

**Funnel 1 RF:**	150.0 Vpp	**Funnel 2 RF:**	200.0 Vpp
**isCID Energy:**	0.0 eV	**Hexapole RF:**	50.0 Vpp
**Ion Energy:**	4.0 eV	**Low Mass:**	90.0 m/z
**Collision Energy:**	7.0 eV	**Pre Pulse Storage:**	5.0 µs
**Stepping:**	On	**Mode:**	Basic
**Collision RF from:**	550.0 Vpp	**Collision RF to:**	550.0 Vpp
**Transfer Time from:**	80.0 µs	**Transfer Time to:**	80.0 µs
**Timing from:**	50 %	**Timing to:**	50 %
**Collision Energy from:**	100 %	**Collision Energy to:**	250 %
**Timing from:**	50 %	**Timing to:**	50 %

MS/MS

**Auto MS/MS:**	Off

MRM

**MRM:**	Off

isCID

**isCID (MS-MS/MS):**	Off

bbCID

**bbCID (MS-MS/MS):**	Off

Segment 2
0.02 .... 0.3 min

Main

**Polarity:**	Positive	**Scan Mode:**	MS
**Mass Range from:**	110 m/z	**Mass Range to:**	1300 m/z
**Rolling Average:**	Off	**Rolling Average No.:**	2
**Spectra rate:**	8.00 Hz	**View:**	Expert

Mode

**Save Spectra:**	Line and Profile Spectra	**Line Spectra Calculation:**	Use Maximum Intensity
**Absolute Threshold (per 1000 sum.):**	25 cts.	**Peak Summation Width:**	3 pts.
		**Focus Active:**	Off
**Mark as Calibration Segment:**	On		

Source

**Source:**	ESI		
**End Plate Offset:**	500 V	**Capillary:**	4500 V
**Nebulizer:**	2.2 Bar	**Dry Gas:**	10.0 l/min
**Dry Temp:**	220 °C	**Divert Valve:**	Source 1-2

Tune

**Funnel 1 RF:**	150.0 Vpp	**Funnel 2 RF:**	200.0 Vpp
**isCID Energy:**	0.0 eV	**Hexapole RF:**	50.0 Vpp
**Ion Energy:**	4.0 eV	**Low Mass:**	90.0 m/z
**Collision Energy:**	7.0 eV	**Pre Pulse Storage:**	5.0 µs
**Stepping:**	On	**Mode:**	Basic
**Collision RF from:**	550.0 Vpp	**Collision RF to:**	550.0 Vpp
**Transfer Time from:**	80.0 µs	**Transfer Time to:**	80.0 µs
**Timing from:**	50 %	**Timing to:**	50 %
**Collision Energy from:**	100 %	**Collision Energy to:**	250 %
**Timing from:**	50 %	**Timing to:**	50 %

MS/MS

**Auto MS/MS:**	Off

MRM

**MRM:**	Off

isCID

**isCID (MS-MS/MS):**	Off

bbCID

**bbCID (MS-MS/MS):**	Off

Segment 3
0.3 .... unlimited min

Main

**Polarity:**	Positive	**Scan Mode:**	Auto MS/MS
**Mass Range from:**	110 m/z	**Mass Range to:**	1300 m/z
**Rolling Average:**	Off	**Rolling Average No.:**	2
**Spectra rate:**	8.00 Hz	**View:**	Expert

Mode

**Save Spectra:**	Line and Profile Spectra	**Line Spectra Calculation:**	Use Maximum Intensity
**Absolute Threshold (per 1000 sum.):**	25 cts.	**Peak Summation Width:**	3 pts.
		**Focus Active:**	Off
**Mark as Calibration Segment:**	Off		

Source

**Source:**	ESI		
**End Plate Offset:**	500 V	**Capillary:**	4500 V
**Nebulizer:**	2.2 Bar	**Dry Gas:**	10.0 l/min
**Dry Temp:**	220 °C	**Divert Valve:**	Waste 1-6

Tune

**Funnel 1 RF:**	150.0 Vpp	**Funnel 2 RF:**	200.0 Vpp
**isCID Energy:**	0.0 eV	**Hexapole RF:**	50.0 Vpp
**Ion Energy:**	4.0 eV	**Low Mass:**	90.0 m/z
**Collision Energy:**	7.0 eV	**Pre Pulse Storage:**	5.0 µs
**Stepping:**	On	**Mode:**	Basic
**Collision RF from:**	550.0 Vpp	**Collision RF to:**	550.0 Vpp
**Transfer Time from:**	80.0 µs	**Transfer Time to:**	80.0 µs
**Timing from:**	50 %	**Timing to:**	50 %
**Collision Energy from:**	100 %	**Collision Energy to:**	250 %
**Timing from:**	50 %	**Timing to:**	50 %

MS/MS

**Auto MS/MS:**	On		
**Precursor Ion List:**	Exclude	**Cycle Time:**	0.5 sec
**Threshold (per 1000 sum.) Absolute:**	400 cts	**Active Exclusion:**	On
		**Exclude after:**	3 Spectra
		**Reconsider Precursor:**	On
**Release after:**	0.20 min.	**Smart Exclusion:**	Off
**if Curent Intens./Prev. Intens.:**	1.8		
**Smart Exclusion:**	2 x		

Exclude Mass List

Mass Range Start	Mass Range End	
102.08	102.18	1
621.98	622.08	2
643.96	644.06	3
659.94	660.04	4

Auto MS/MS Preference

**Preferred Range:**	Off		
**Preferred Range Low:**	2	**Preferred Range High:**	5
**Exclude Singly:**	Off	**Exclude unknown:**	Off
**Group Length:**	3	**Strict Active Exclusion:**	Off
**Sort Precursors by:**	Intensity	**Preferred mass list:**	Empty list

Auto MS/MS Multi CE

Auto MS/MS Multi CE: Off

SILE

SILE: Off

CID

Fallback Charge State: 1 z

Isolation + Fragmentation List

Type	Mass [m/z]	Width [m/z]	Collision Energy [eV]	Charge State	
Base	100.00	4.00	20.0	1	1
Base	500.00	5.00	20.0	1	2
Base	1000.00	6.00	20.0	1	3
Base	1300.00	8.00	30.0	1	4

CID Acquisition

**Acquisition:**	On	**Spectra Rate MS:**	8.00 Hz
**MS/MS low (per 1000 sum.):**	10000.0 cts.	**MS/MS low:**	1600 x
**MS/MS high:**	100000.0 cts.	**MS/MS high:**	800 x
**Total Cycle Time Range:**	n/a sec	**Absolute Threshold :**	n/a cts.

MRM

MRM: Off

isCID

isCID (MS-MS/MS): Off

bbCID

bbCID (MS-MS/MS): Off
